# Halted Lymphocyte Egress via Efferent Lymph Contributes to Lymph Node Hypertrophy During Hypercholesterolemia

**DOI:** 10.3389/fimmu.2019.00575

**Published:** 2019-03-27

**Authors:** Meng Hwee Daniel Tay, Swee Yeng Jason Lim, Yew Fai Ivan Leong, Chung Hwee Thiam, Kar Wai Tan, Federico Tesio Torta, Pradeep Narayanaswamy, Markus Wenk, Véronique Angeli

**Affiliations:** ^1^Immunology Programme, Department of Microbiology and Immunology, Life Science Institute, Yoon Loo Lin School of Medicine, National University of Singapore, Singapore, Singapore; ^2^Department of Biochemistry, Life Science Institute, SLING-Singapore Lipidomics Incubator, Yoon Loo Lin School of Medicine, National University of Singapore, Singapore, Singapore

**Keywords:** lymph node, lymphatic vessel, lymphocyte egress, mouse model, dyslipidemia

## Abstract

Dyslipidemia is a central component of atherosclerosis and metabolic syndrome linked to chronic inflammation and immune dysfunction. Previously, we showed that hypercholesterolemic apolipoprotein E knock out (apoE^−/−^) mice exhibit systemic effects including skin inflammation and hypertrophic lymph nodes (LNs). However, the mechanisms accounting for LN hypertrophy in these mice remain unknown. Here, we show that hypercholesterolemia led to the accumulation of lymphocytes in LNs. We excluded that the increased number of lymphocytes in expanded LNs resulted from increased lymphocyte proliferation or entry into those LNs. Instead, we demonstrated that the egress of lymphocytes from the enlarged LN of apoE^−/−^ mice was markedly decreased. Impairment in efferent lymphatic emigration of lymphocytes from LNs resulted from an aberrant expansion of cortical and medullary sinuses that became hyperplastic. Moreover, CCL21 was more abundant on these enlarged sinuses whereas lymph levels of sphingosine 1 phosphate (S1P) were decreased in apoE^−/−^ mice. Normal LN size, lymphatic density and S1P levels were restored by reversing hypercholesterolemia. Thus, systemic changes in cholesterol can sequester lymphocytes in tissue draining LNs through the extensive remodeling of lymphatic sinuses and alteration of the balance between retention/egress signals leading to LN hypertrophy which subsequently may contribute to poor immunity. This study further illustrates the role of lymphatic vessels in immunity through the regulation of immune cell trafficking.

## Introduction

Lymph nodes (LNs) are highly specialized organs that play an essential role in immune priming and function. LN comprises of three main regions namely the cortex, paracortex, and the medulla. Lymph enters the LN via the afferent LVs and reaches the subcapsular sinuses first before it drains through the cortical sinuses in the cortex region, and into the medullary sinuses in the medulla region, and then eventually exits the LN via efferent lymphatic vessels. Protein, lipid, antigens, microorganisms, and immune cells such as lymphocytes and dendritic transported via the lymph enter the subcapsular sinus of the LN. Subcapsular and medullary sinuses are directly connected and thus lymph can pass through LN without filtering through the cortex (1). In most cases, lymphocytes enter the LN via the high endothelial venules (2), except for a small proportion of memory lymphocytes that enter via the lymphatics (3, 4). Activated and naïve lymphocytes in the LN matrix would eventually need to enter the sinuses so that they can be transported by the efferent lymph to reach the effector sites or return back to the circulation for immune surveillance, respectively. Lymphatic endothelial cells (LECs) in the cortical sinuses control lymphocyte trafficking within the LN by accumulating lymphocytes for further transit to medullary sinuses (5). Furthermore, lymphocytes can migrate from the lymphatic sinuses back to the LN parenchyma (5). Lymph borne lymphocytes can also be passively collected into the peripheral medullary sinuses, and then enter the LN parenchyma in a CCR7-independent manner by moving into adjacent peripheral medullary cords (6). The medulla is formed of a three-dimensional labyrinthine structure of sinus channels starting as cortical sinusoids and expand to become wider medullary sinuses that finally drain collectively into the efferent lymphatic vessel (7). In addition to cortical sinuses, medullary sinuses have been proposed as exit routes for the egress of lymphocytes from LNs (5, 8, 9). Lymphocyte egress are governed by mechanisms controlling the entry of lymphocytes into the efferent lymphatic vessels including the regulation of CCR7 and sphingosine-1-phosphate receptor-1 expression (10–12), or those regulating lymphatic endothelial barriers (13, 14). It is well-established that lymphocyte egress from LNs via cortical and medullary sinuses is dependent on signals generated by lymph-borne S1P (8, 9, 15) produced by LECs via S1P kinase 1 kinase (16). S1P levels are low in LN parenchyma but high in lymph fluid thus creating a gradient. This S1P gradient guides T cells exhibiting decreased CCR7/CCL21-retention signals from LN parenchyma into medullary and cortical sinuses and ultimately facilitates T cell egress (10). Thus, the structural integrity and functions of these lymphatic sinuses have to be maintained as they are not only just passive carrier of lymph, but active player in the regulation of lymphocyte egress.

Local immune responses and inflammation are associated with alterations in the trafficking of lymphocytes through activated LNs. Indeed, the entry of lymphocytes into the draining LNs is increased whereas their exit into the efferent lymph is transiently blocked from few hours to days depending on the nature of the antigen and inflammation (17–20). In contrast to our knowledge about the mechanisms controlling lymphocyte retention within the inflamed LN after the initiation of an immune response (8, 21, 22), little is known about those reestablishing lymphocyte egress to steady-state levels. We showed previously that the expansion of cortical and medullary sinuses in the stimulated LNs at later stages of inflammation contribute to the reestablishment of lymphocyte egress rates (23). Reestablishment of steady-state egress from inflamed LNs may help to content the enhanced lymphocyte entry into LNs when inflammation is prolonged. This may prevent these LNs from becoming a “sink” that may compromise efficient lymphocyte recirculation and the timely induction of a suitable immune response (24, 25). This raises the possibility that LN hypertrophy and hyperactivation occurring with autoimmune and chronic inflammatory diseases may arise when lymphocytes fail to emigrate from the inflamed LNs in a timely manner. Notably, we and others reported in mice that hypercholesterolemia associated with atherosclerosis disease leads to systemic effects including skin inflammation, hypertrophic LNs, and compromised immunity (26–33). However, how hypercholesterolemia lead to LN hypertrophy in still remains an open question. Here, we explored how lymphocyte trafficking and LN structure and function are affected by hypercholesterolemia that occurs in mice lacking apoE (apoE^−/−^). The study reveals an accumulation of lymphocytes in enlarged skin draining LNs which results from a severe blockade of their egress rather than increased lymphocyte proliferation or entry within the enlarged LNs. We further show that hypercholesterolemia induces an aberrant remodeling of the cortical and medullary sinuses and an imbalance between retention and egress signals that account for the impaired lymphocyte egress in dyslipidemic mice.

## Methods

### Animals

Male CD45.2 C57BL/6 mice deficient in apolipoprotein E (apoE^−/−^ mice) and low-density lipoprotein receptor (Ldlr^−/−^), CD45.2 wild type C57BL/6 (WT), and CD45.1 C57BL/6 WT were obtained from The Jackson Laboratory (Bar Harbor, ME). All mice were given a chow diet (18% protein and >5% fat, Harlan Teklad, Madison, WI) until 6 weeks of age. At 6 weeks of age, the diet was switched to a high fat and cholesterol rich diet containing 21.2% fat and 0.2% cholesterol (Harlan Teklad) until sacrifice. In some experiments, 13–16 weeks old male apoE^−/−^ and WT mice fed a high fat diet were treated daily with ezetimibe (5 mg/kg; Kemprotec) or corn oil (vehicle) via oral gavage corn oil for 12 weeks prior sacrifice as previously described (34). All mice were housed under specific pathogen free conditions with unrestricted access to food and water in the animal housing unit of the National University of Singapore. All studies were performed under protocols approved by the Institutional Animal Care and Use Committee (IACUC) of the National University of Singapore.

### Adoptive Cell Transfers

For adoptive transfers, CD45.2 recipient mice were intravenously injected with 2 × 10^7^ of CD45.1 spleen and LN cells. In short term homing experiments to assess lymphocyte entry into skin draining LNs (axillary and brachial), CD45.1, or CD45.2 cells were adoptively transferred into CD45.2 or CD45.1 recipient mice, respectively and allowed to equilibrate for 2.5 h before sacrifice. At this time point, entry from the peripheral blood into skin draining LNs directly determined the CD45.1 cell numbers within recipient LNs (23, 35). In experiments where donor lymphocytes from CD45.1 WT and CD45.2 apoE^−/−^ mice were co-transferred, lymphocytes were labeled with carboxyfluorescein succinimidyl ester (CFSE). To assess lymphocyte egress from skin draining LN (axillary and brachial), CD45.1, or CD45.2 cells were adoptively transferred into CD45.2 or CD45.1 recipient mice, respectively and allowed to equilibrate for 24 h. Following equilibrium, half of the mice were sacrificed (T_0_) while the other half of the mice were treated with anti-CD62L antibody and sacrificed 20 h after administration (T_20_). Intraperitoneal administration of anti-CD62L antibody at a dose of 100 μg per mouse (clone Mel-14 hybridoma from American Type Culture Collection) blocked further entry of circulating lymphocytes into LNs without affecting lymphocyte egress from LNs (8, 10). Therefore, the population of transferred lymphocytes remaining within skin draining LNs at T_20_ compared to T_0_ will be a measure of lymphocyte egress from LNs (23).

### Spleen, LN, and Lymph Cell Suspensions

Cell suspensions from skin draining LNs and spleens were prepared by mechanical disruption. For quantification of LECs by flow cytometry, LNs were first digested with 4 mg/mL collagenase IV (Roche) in calcium and magnesium free HBSS at 37°C for 45 min with gentle agitation. After 45 min, EDTA was added to the digestion mixture to a working concentration of 10 mM and the lymph nodes were further digested for 5 min at 37°C in a final digestion step. For lymph cell collection, mice were fasted overnight prior sacrifice and lymph was collected as described by Matloubian et al. (36). The peritoneal cavity of anesthetized mice was exposed ventrally and the cysterna chili identified. Lymph was subsequently drawn from the cysterna chili with the use of extended length gel-loading pipette tips (Neptune Scientific).

### Flow Cytometry Analysis

Flow cytometric analysis of skin draining LN (brachial and axillary) cell suspensions stained for CD45, podoplanin and CD31 allowed differentiation and quantification of LECs (CD45^−^, CD31^+^, podoplanin^+^) (23). Antibodies used included the following: rat anti-mouse CD31 (Serotec) detected with anti-rat-APC, hamster anti-mouse podoplanin (clone 8.1.1; Developmental Studies Hybridoma Bank, University of Iowa, Iowa City, IA) detected with anti-hamster PE and PerCPcy5.5—conjugated anti-mouse CD45.2 (BD Biosciences). FACS analysis was also employed to quantify congenic transferred lymphocytes T and B cell populations in LNs, and lymph. Antibodies used included the following: FITC or PerCPCy5.5—or Pacific Blue conjugated anti-B220, APC-conjugated anti-CD3e, PerCPCy5.5-conjugated anti-CD45.2, biotin–conjugated anti-CD45.1 revealed with Streptavidin-PE. Live and dead cells were discriminated during flow cytometry using a LIVE/DEAD® Fixable Aqua Dead Cell Stain Kit (Molecular Probes, Invitrogen). Cell counts were determined during flow cytometry using Count Bright® Absolute Counting Beads (Molecular Probes, Invitrogen). FACS analysis was performed using a CyAn ADP Analyzer (Beckman Coulter) and data were analyzed with Flowjo software (Treestar).

### Immunohistochemistry

Skin draining LNs (axillary and brachial) were either freshly embedded in tissue freezing medium or fixed overnight in 2% paraformaldehyde/30% sucrose solution at 4°C and embedded in tissue freezing medium. 6–8 μm thick cryostat sections were cut for imaging by the fluorescence microscope. Primary antibodies used included biotinylated or purified anti-B220 (eBiosciences), anti-TCRβ (BD Biosciences), anti-LYVE-1 (Upstate), anti-CD31 (Serotec), anti-Ki67 (Dako), anti-CCL21 (R&D Systems), anti-collagen type IV (Cosmo Bio), FITC-conjugated anti-CD169 (Serotec), biotinylated anti-CD45.1 (eBiosciences) antibodies. Secondary antibodies used included Dylight647-conjugated streptavidin, Cy2 or Cy3-conjugated anti-rat IgG, Dylight647 or Dylight549-conjugated anti-armenian hamster IgG, Dylight 647-conjugated anti-goat IgG and Cy2, Cy3, or Dylight647-conjugated anti-rabbit IgG antibodies (Jackson Immunoresearch). Endogenous avidin and biotin were quenched using the Avidin/Biotin blocking kit (Vector Laboratories).

### Microscopy, Image Analysis, and Lumen Area Measurements

To anatomically locate the lymphatic sinuses in the paracortex and medulla of the skin draining LNs images of LN sections stained for LYVE-1 and B220 were captured with a fluorescence microscope (Axio imager.Z1, Axiocam HRM camera; Carl Zeiss Micro Imaging, Inc., Jena, Germany). Lymphatics were identified in cortical or medullary sinuses if they were found in the LN paracortex or medulla and to contain B cells within their lumen (23). To determine lumen area of lymphatic sinuses, images were acquired from 6 representative LN sections per mouse. Within each section, the area of 15 lymphatic vessels in the cortex region and 10 lymphatic vessels in the medulla region were quantified. Therefore, a total of 90 cortical and 60 medullary sinuses were sampled per mouse. Lumen area was obtained by using the “measure” tool of the Axiovision software (version 4.8; Carl Zeiss Micro Imaging, Inc.).

### S1P Measurement by Liquid Chromatography-Mass Spectrometry

The lymph samples were centrifuged at 14,000 g for 10 min at 4°C and the supernatant was obtained for subsequent S1P extraction. Five microliter of lymph was mixed with 5 μl ISTD and 90 μl methanol. The samples were sonicated for 30 min at room temperature and centrifuged at 14,000 g for 10 min. The supernatant was recovered and 10 μl of TMS-Diazomethane (2M in hexane) was added. The sample incubated for 20 min at room temperature under gentle mixing at 750 rpm. The reaction was stopped by adding 1 μl of acetic acid. The derivatized samples were dried in speedvac and reconstituted in 100 μl of mobile phase before injecting 1 μl of sample into the LC-MS system. All solvents for LC-MS analysis were LC-MS grade and were purchased from Fisher Scientific and Merck Millipore. Lipid standards: isotope labeled standard D-erythro-Sphingosine-1-phosphate (13C2D2–S1P,) were purchased from Toronto Research Chemicals. All LC-MS/MS experiments were performed using Agilent 1200 series HPLC-Chip systems connected to the Agilent 6490 QQQ mass spectrometer as described in our previous report (37). A customized HILIC-chip containing Amide-80 stationary phase (Tosoh Bioscience, LLC. Montgomeryville, PA, 5 Nm particle size, 80 Å pore size) was used for the chromatographic separation, including a 160 nl trapping column and a 75 Nm × 150 mm analytical column (Agilent Technologies Corp., Santa Clara CA). Solvents used for HILIC HPLC: 50% acetonitrile in water containing 25 mM ammonium formate pH 4.6 (solvent A), 95% acetonitrile containing 25 mM ammonium formate pH 4.6. The pH value was adjusted with formic acid. Analytes were eluted with the following gradient: 100% B from 0 to 1.5 min, 40% B from 1.5 to 8.5 min, 30% B from 8.5 to 10.5 min, 0% B from 11.5 to 13.0 min, 100% B from 13.1 to 19 min. The chip cube was operated with back flush mode and samples were injected through the enrichment column at 4 μl/min. The valve was switched 1.5 min after injection to place the enrichment column in line with the analytical column at a flow rate of 400 nl/min. Murine lymph samples were spiked with known amounts of internal standard (S1P-13C2D2). The Agilent 6490 triple quadrupole (QQQ) mass spectrometer was operated in positive mode for MRM where the CID fragment at m/z 60 was used as a “quantifier” and m/z 113 was used as a “qualifier.” These ions were present after fragmentation of all species. Quantification was performed according to the internal standard method, comparing peak areas of the endogenous S1P extracted with MassHunter Quant (Agilent) to the ISTD peak.

### CCL21 ELISA

Skin draining LNs were harvested and homogenized in lysis buffer (RIPA buffer, Sigma Chemicals) with a protease inhibitor cocktail (Roche Diagnostics). Homogenates were centrifuged for 10 min at 4°C at 14,000 g, and supernatants were assayed using commercial CCL21 (R&D Systems) ELISA kits as per manufacturer's protocols.

### Quantitative RT-PCR

Total RNA from skin draining LNs was homogenized and extracted using TRIZOL® reagent (Invitrogen) and NucleoSpin® RNA II kit (Macherey-Nagel). First strand cDNA was synthesized using TaqMan® Reverse Transcription Reagents (Applied Biosystems). Real-time PCR were performed using iTaq^TM^ SYBR® Green supermix with ROX (Biorad) on a 7500 Real-Time PCR System (Applied Biosystems). Expression of genes of interest expression was normalized to the expression of GAPDH. The following primers were used: *Sphk1*, forward 5′-AACTTGACTGTCCATACCTGGTTC-3′ and reverse 5′-CACATACCATCAGCTCTCCATCC-3′; *Sgpl1*, forward, 5′-CCTGTTGGGCCGCCTTGATGC-3′ and reverse 5′-AAATTCCACCCCTTAGC-3′.

### Statistical Analysis

Statistical analysis was performed with Prism 5 (Graph-Pad Software, Inc.). All values were expressed as the mean of *n* samples ± SD. Statistical significances were determined using the unpaired two-tailed *t*-test. Whenever more than two groups were compared, the one-way ANOVA test with Bonferroni's post-test was applied. For all tests, a *p* < 0.05 was considered significant.

## Results

### Lymphocytes Accumulate in Hypertrophic LN From apoE^−/−^ Mice

**Figure 1:**
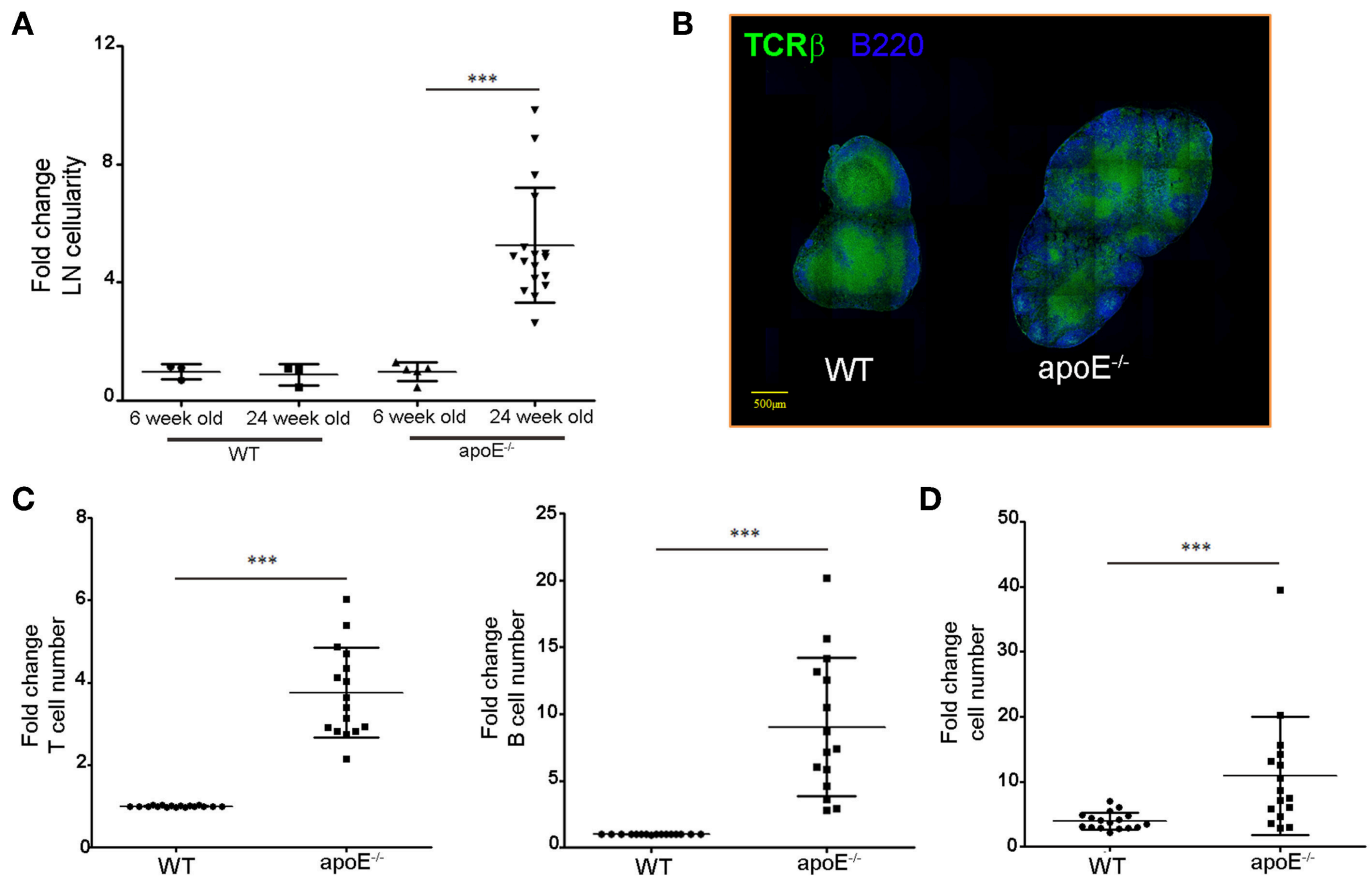
Dyslipidemia is associated with LNs hypertrophy. **(A)** Lymph node cellularity was determined in 6 and 24 weeks old WT and apoE^−/−^ mice and was expressed as fold change over WT mice. Data is pooled from four independent experiments with 4–5 mice per group in each experiment. **(B)** Immunoreactivity for B220 and TCRβ was examined in LN sections from WT and apoE^−/−^ mice at 22 to 28 weeks of age. **(C)** T and B cell numbers were examined by flow cytometry and results were expressed as fold change over WT mice. **(D)** The fold change in T and B cells over WT mice was examined. ***p < 0.0005.

Consistent with our previous report (33), the substantial increase in skin draining LN cellularity was evident in 22 to 28 weeks old apoE^−/−^ mice fed a diet rich in fat and cholesterol compared to age-matched WT mice but not in 6 week old apoE^−/−^ mice (Figures 1A,B). LN hypertrophy was also observed in Ldlr^−/−^ mice, another hypercholesterolemic mouse model (Supplemental Figure 1). Flow cytometry analysis revealed T and B cells accumulation in the enlarged LNs of apoe^−/−^ mice (Figure 1C) with a proportionally greater increase in B cells compared to T cells (Figure 1D). The number of CD4^+^ and CD8^+^ T cells increased in hypertrophic LN of *Apoe*^−/−^ mice but the ratio of CD4^+^/CD8^+^ T cells was similar to WT mice (data not shown).

### Lymphocyte Proliferation and Entry Within Expanded LNs From apoE^−/−^ Mice Are Not Altered

**Figure 2:**
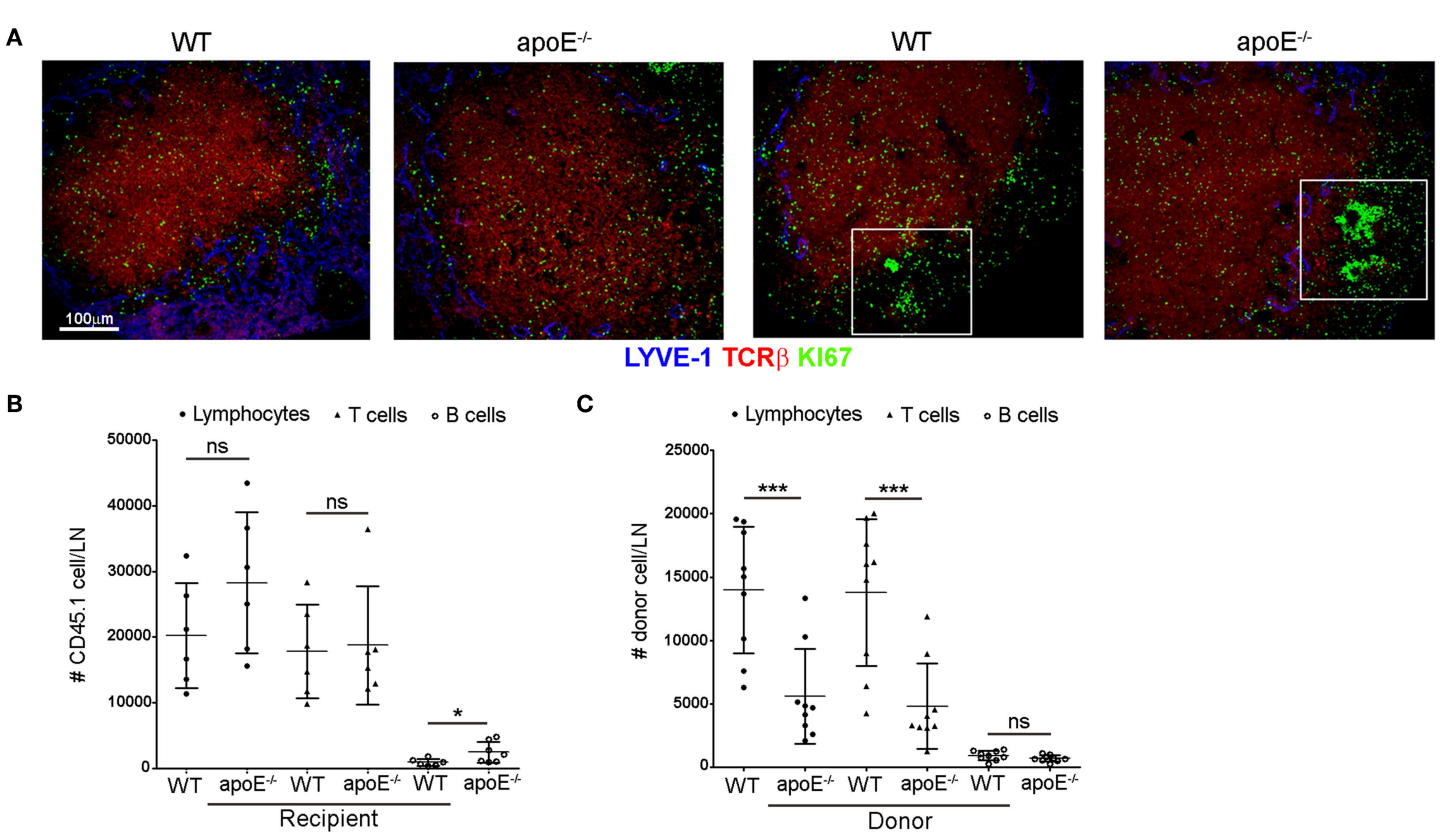
Proliferation and entry of lymphocytes in apoE^−/−^ mice are not affected by dyslipidemia. **(A)** LNs sections from 22 to 28 weeks old WT and apoE^−/−^ mice were double stained for TCRβ and Ki-67. Box demarcates B cell follicle **(B)** Short term homing experiments were performed to assess the entry of CD45.1 WT lymphocytes, T or B cells into LNs from CD45.2 WT and apoE^−/−^ recipient mice. **(C)** The entry of CD45.2 CFSE-labeled WT or apoE^−/−^ lymphocytes, T or B cells into WT LNs was examined. *n* = 9 mice per group. *p < 0.05; ***p < 0.0005.

Accumulation of lymphocytes in an activated LN may result from increased proliferation within LN, increased entry of lymphocytes from the blood into the LN or, conversely, a decreased efferent lymphatic emigration from the enlarged LN. Therefore, we sought to determine which of these possibilities could account for LN hypertrophy in apoE^−/−^ mice. We excluded the possibility of lymphocyte proliferation contributing to the LN hypertrophy in apoE^−/−^ mice since we did not detect any obvious differences for proliferative marker Ki-67 in apoE^−/−^ and WT LN sections co-stained with TCRβ or B220 to detect T cells and B cells, respectively (Figure 2A). To determine whether lymphocyte trafficking into the enlarged LNs of apoE^−/−^mice is affected, we quantitated lymphocyte entry into LNs in short-term homing assays (23). 2.5 hours after adoptive transfer of CD45.1 WT lymphocytes into CD45.2 apoE^−/−^ or WT mice, CD45.1-transferred T cells accumulated similarly in the LN of WT and apoE^−/−^ recipient mice (Figure 2B) whereas the number of transferred B cells was increased in apoE^−/−^ LN compared to WT LN. Despite subtle differences in T and B cell trafficking, the overall entry of CD45.1-transferred lymphocytes into apoE^−/−^ and WT LN was similar (Figure 2B), indicating that lymphocyte entry into the hypertrophic LN of *Apoe*^−/−^ mice was not augmented. To substantiate these findings, we performed reverse adoptive transfer experiments. Lymphocytes, T or B cells isolated from CD45.1 WT and CD45.2 apoE^−/−^ mice were labeled with CFSE and co-transferred into CD45.1 WT recipient mice. Unexpectedly, we found that the entry of apoE^−/−^ lymphocytes into WT LN was decreased compared to WT lymphocytes (Figure 2C). Further analysis revealed that apoE^−/−^ T cell entry was decreased whereas input of apoE^−/−^ B cells into WT LN was comparable to WT B cells. Taken together, these data indicate that lymphocyte accumulation in enlarged LN of apoE^−/−^ mice is not due to increased lymphocyte entry into the LNs.

### The Efferent Lymphatic Emigration of Lymphocytes From LN Is Markedly Blocked in apoE^−/−^ Mice

**Figure 3:**
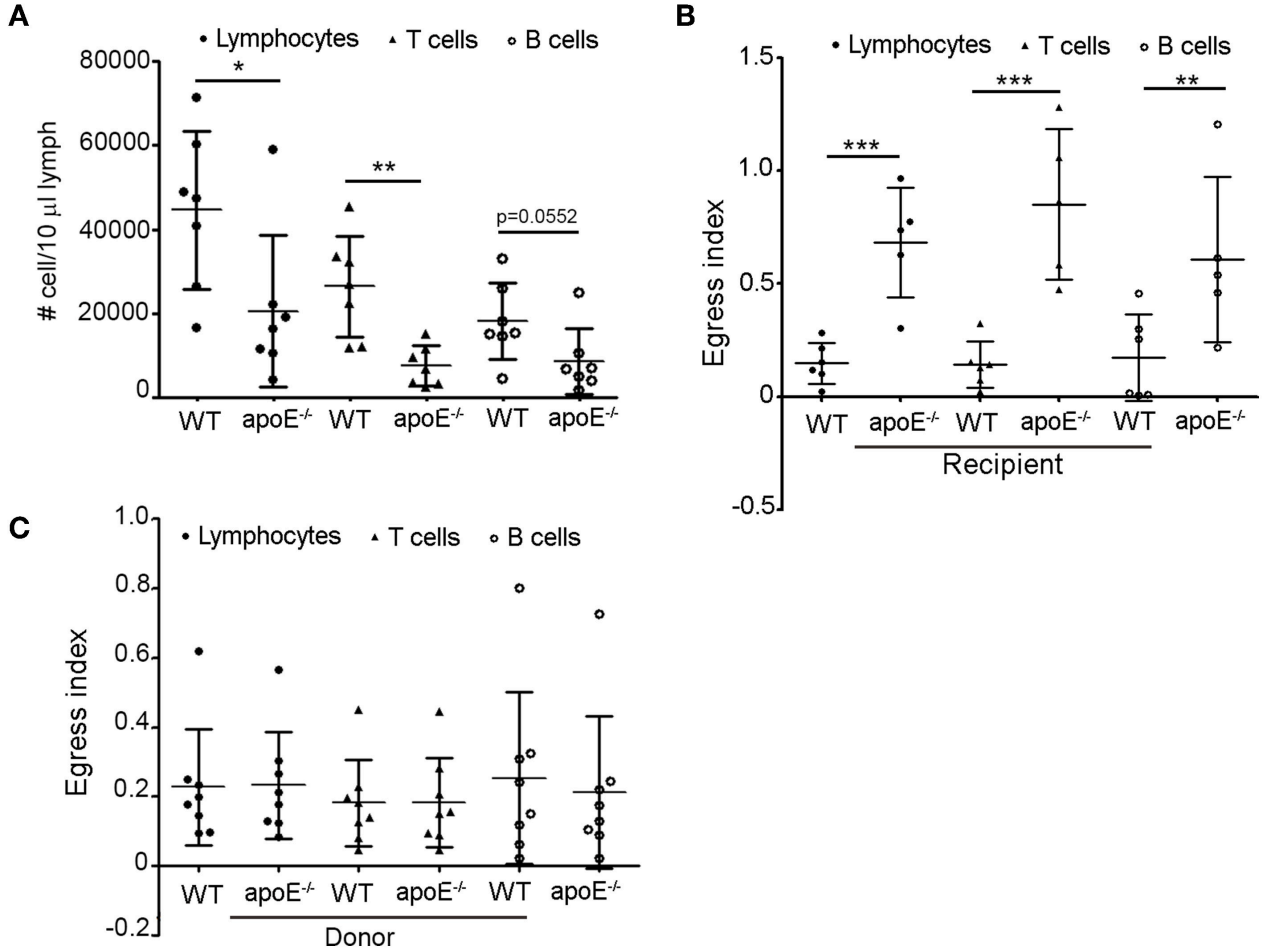
Lymphocyte egress from apoE^−/−^ mice LN is compromised **(A)** Lymphocytes, T and B cell were enumerated in lymph from 22 to 28 weeks old WT and apoE^−/−^ mice by flow cytometry. **(B)** Using lymphocyte adoptive transfer assay, the egress of transferred CD45.1 WT lymphocytes, T or B cells from WT or apoE^−/−^ CD45.2 recipient LNs was assessed. Results were expressed as mean fraction of egressed T cells from LNs. **(C)** The egress of transferred CD45.2 WT or apoE^−/−^ lymphocytes, T or B cells from WT CD45.1 recipient LNs was assessed. Results were expressed as mean fraction of egressed T cells from LNs. *n* = 7–10 mice per group *p < 0.05; **p < 0.005; ***p < 0.0005.

Since we rule out the involvement of lymphocyte entry and proliferation to impact lymphocyte accumulation in LNs of Apoe^−/−^ mice, we hypothesized that the increased number of lymphocytes in skin draining LNs from apoE^−/−^ mice could result from impaired egress of these cells from the enlarged LN via efferent lymphatic vessels. In support of this hypothesis, we found a marked decrease in the number of total lymphocytes, T and B cells in efferent lymph from apoE^−/−^ mice collected at the cysterna chyli (36, 38) compared to WT efferent lymph (Figure 3A). This prompted us to assess the exit of lymphocytes from LN using long-term adoptive transfer assays (23, 36). We first evaluated the capacity of CD45.1 WT donor lymphocytes to egress from CD45.2 WT and apoE^−/−^ LN mice. The data were expressed as mean fraction of egressed cells—defined by dividing the mean CD45.1 T cell that have exited (T_20_) by the mean population of CD45.1 T cells present at baseline (T_0_) (23). This experiment revealed that the egress of WT transferred lymphocytes from hypertrophic apoE^−/−^ recipient LN was severely abrogated compared to WT recipient LN (Figure 3B). This phenomenon was observed for both T and B cell egress. Next, we compared the egress of CD45.2 apoE^−/−^ lymphocytes from CD45.1 WT recipient LN with the egress capacity of CD45.2 WT lymphocytes. No significant difference was observed between the egress index of WT and apoE^−/−^ lymphocyte, T or B cells (Figure 3C). Considering that no other alternative possibilities could account for the greatly increased numbers of lymphocyte in the hypertrophic LN from apoE^−/−^ mice, we conclude that increased lymphocytes accumulation in expanded skin draining LN occurs because less lymphocytes emigrate from those LNs into the efferent lymph.

### Lymphangiogenesis Is Induced in Hypertrophic LNs

**Figure 4:**
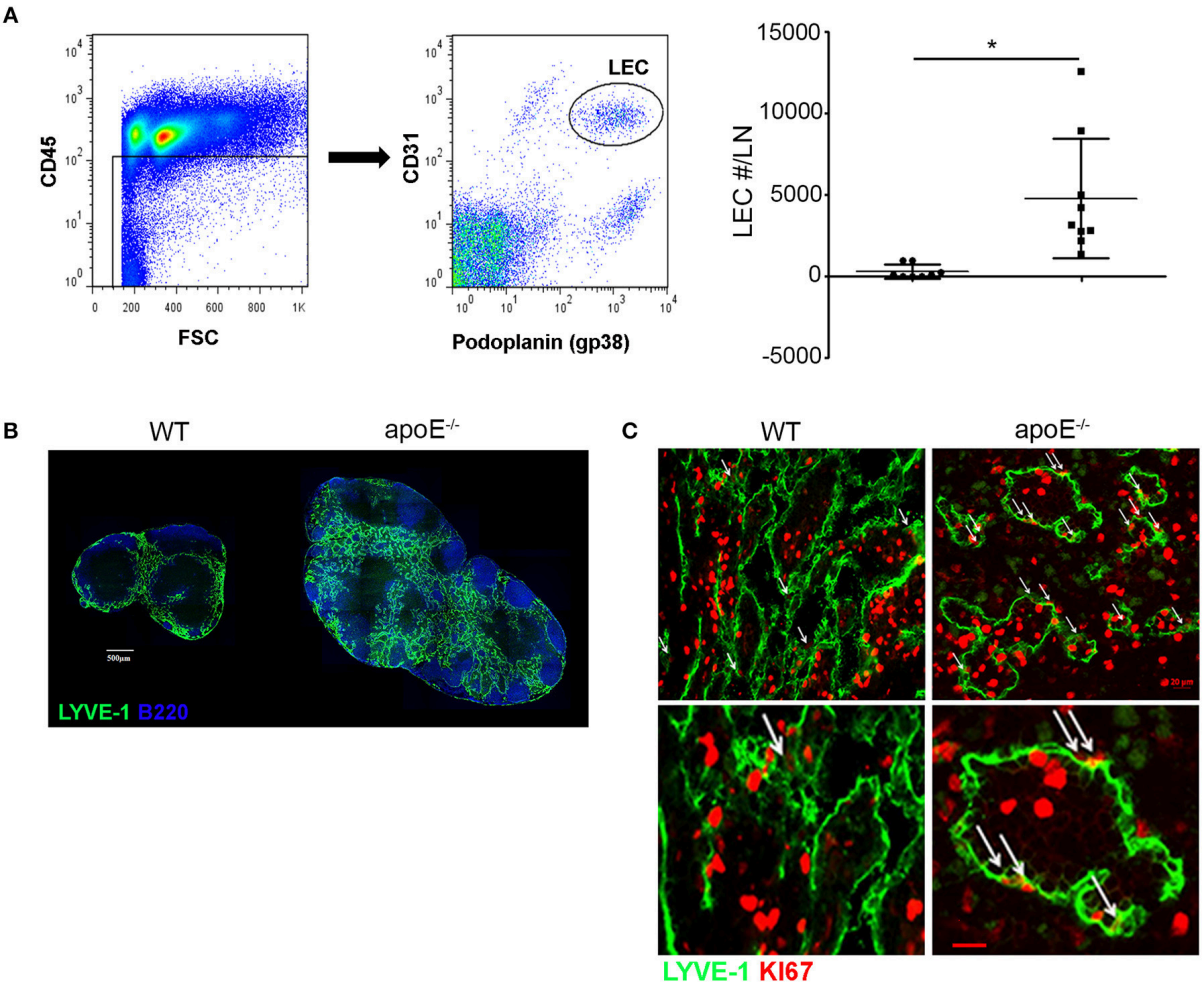
Dyslipidemia induces LN lymphangiogenesis **(B)** LN sections from WT and apoE^−/−^ mice at 22 to 28 weeks of age were stained for B220 and LYVE-1. Images are representative of four independent experiments (*n* = 3–4 mice per group). **(A)** LN cells were stained for CD45, podoplanin and CD31 to identify LECs by FACS analysis. The number of LEC was enumerated in LNs from WT and apoE^−/−^ mice at 22 to 28 weeks of age and expressed as fold change over WT mice. Data is pooled from three independent experiments with 3–5 mice per group in each experiment; *p < 0.05. **(C)** LYVE-1^+^ vessels were analyzed for co-expression of the proliferative marker KI67. Arrows indicate co-localization of lymphatics with Ki67. Ki67^bright^ dividing lymphocytes were also observed. Images are representative of three to four independent experiments (*n* = 3–4 mice per group).

Our long-term adoptive transfer assays indicate that the impaired egress of lymphocytes from apoE^−/−^ hypertrophic LN was not due to intrinsic defects in lymphocytes but rather to environmental changes in the enlarged LN. We hypothesized that the hypercholesterolemic environment in apoE^−/−^ mice induces LN remodeling compromising the egress of lymphocytes, which in turn promotes subsequent LN hypertrophy. As the lymphatic vessels are critical routes for lymphocyte egress from LNs and inflammation has been shown to induce lymphangiogenesis in activated LN such as upon immunization (23, 38), we examined LN lymphangiogenesis in apoE^−/−^ mice. We employed flow cytometry to quantify LECs in LNs (Figure 4A). Hypercholesterolemia in apoE^−/−^ mice stimulated a significant increase in LEC numbers over WT LN (Figure 4B). This marked expansion of lymphatic network in hypertrophic skin draining LNs from apoE^−/−^ mice was also apparent in LN sections immunostained for LYVE-1 which identifies lymphatic sinuses in the medulla and cortex (Figure 4C). Moreover, immunostaining of LN sections for proliferative marker Ki67 revealed that the lymphatic expansion resulted from proliferation of pre-existing lymphatics (Figure 4D).

### Expanded Cortical and Medullary Sinuses Are Overly Dilated in Hypertrophic LN

**Figure 5:**
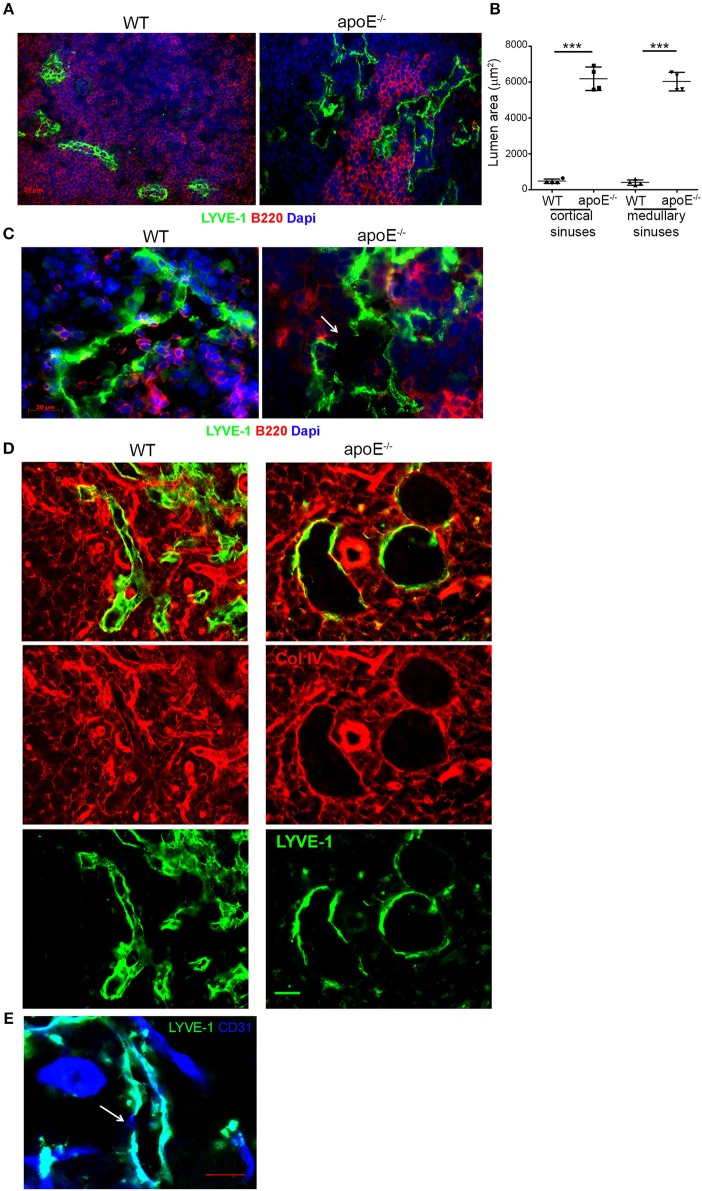
The extended lymphatic network in apoE^−/−^ mice LNs exhibits structural abnormalities. **(A)** Immunoreactivity for LYVE-1 was examined in LN sections from WT and apoE^−/−^ mice at 22–28 weeks of age. **(B)** Lumen area of cortical and medullary sinuses was determined on LN sections from 22–28 week-old WT and apoE^−/−^ mice. *n* = 4 mice per group; ***p < 0.0005 **(C)** Immunoreactivity for LYVE-1 and B220, **(D)** LYVE-1 and collagen (Col) type IV or **(E)** LYVE-1 and CD31 was examined in LN sections from apoE^−/−^ mice at 22–28 weeks of age. Images are representative of 3–4 independent experiments (*n* = 3–5 mice per group). Arrows indicate loss of LYVE-1 expression.

We next investigated whether the extensive expansion of cortical and medullary sinuses observed in apoE^−/−^ LN was accompanied by any morphological changes in the sinuses. A close-up examination using LYVE-1 revealed that majority of the cortical and medullary sinuses in hypertrophic LN from apoE^−/−^ mice were notably dilated with open lumens compared to those in WT LN with more partially collapsed state (Figure 5A). This was further supported by the marked increase in lumen area of cortical and medullary sinuses in apoE^−/−^ mice LN compared to WT control LN (Figure 5B). Moreover, the dilated sinuses were more irregular and tortuous with occasional focal absence of LYVE-1 expression (Figure 5C). These morphological changes resemble those described in certain tumor-associated vessels having regions lacking endothelial cell markers and termed “mosaic vessels” (39). Additional co-staining of LN sections with LYVE-1 and collagen type IV or CD31 that identify the basement membrane and endothelial cells of sinuses, respectively, confirmed the focal absence of LYVE-1 expression in the cortical and medullary sinuses of apoE^−/−^ mice compared to WT mice although collagen type IV (Figure 5D) and CD31 (Figure 5E) remained intact in those regions. These data indicated extensive structural changes in the LN cortical and medullary sinuses from dyslipidemic mice.

### The Balance Between Retention and Egress Signals Is Altered in Hypetrophic LNs

**Figure 6:**
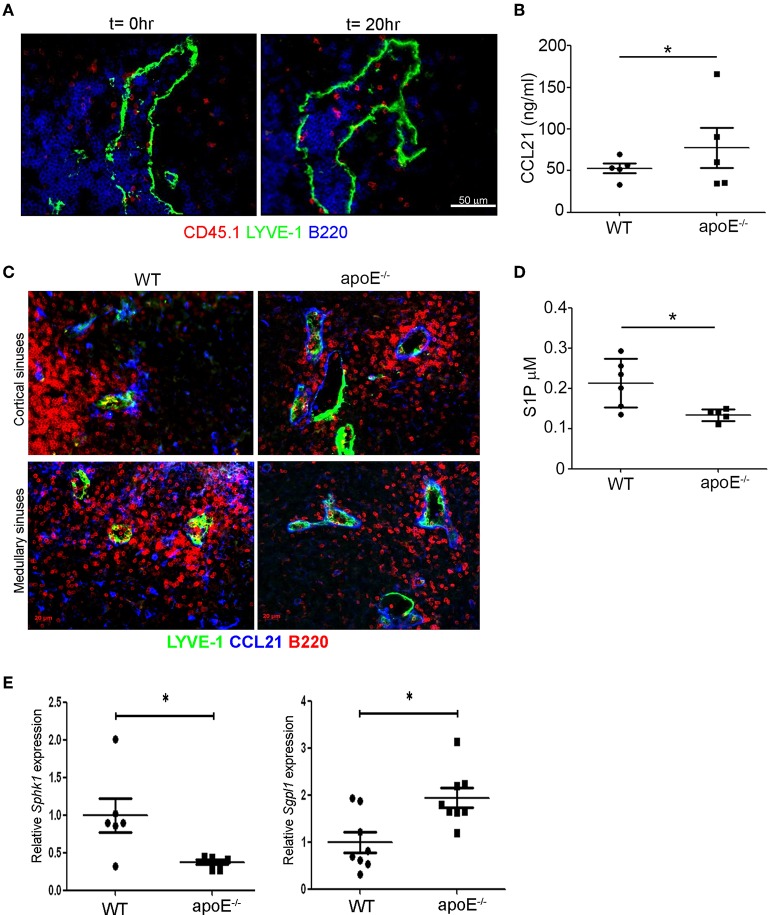
The balance between retention and exit signals is altered in apoE^−/−^ mice LNs. **(A)** Immunoreactivity for LYVE-1, B220, and CD45.1 was examined on LN sections from long-term adoptive transfer at *t* = 0 and *t* = 20 h to determine the localization of CD45.1 transferred lymphocytes in enlarged LNs from apoE^−/−^ mice. Images are representative of three to four independent experiments (*n* = 3–4 mice per group) **(B)** CCL21 protein content was analyzed in homogenates of 22 to 28 weeks-old WT and apoE^−/−^ mice LNs by ELISA. **(C)** Immunoreactivity for CCL21 and LYVE-1 was examined in LN sections from 22 to 28 weeks-old WT and apoE^−/−^ mice. Images are representative of three to four independent experiments (*n* = 3–5 mice per group) **(D)** S1P content was analyzed in efferent lymph of 22 to 28 weeks-old WT and apoE^−/−^ mice by mass spectrometry. **(E)** Quantitative RT-PCR analysis was performed on whole LN RNA to examine expression of *Sph1k* and *Spgl1* in WT and apoE^−/−^ mice at 22 to 28 weeks of age. *n* = 6–10 mice per group; *p < 0.05.

We next investigated whether these structural alterations may compromise the entry of lymphocytes into the sinuses. To address this question, we stained LN sections from long-term adoptive transfer experiments for CD45.1 at T_0_ and T_20h_ to identify the WT donor lymphocytes in LNs from WT and apoE^−/−^ mice. While we failed to capture transferred lymphocytes within collapsed sinuses in WT LN (data not shown), numerous transferred lymphocytes were detected within the dilated sinuses in apoE^−/−^ LN (Figure 6A). Thus, although the lymphocytes were able to migrate into the exit structures of enlarged LN they failed to be captured by the lymph flow and transported to the efferent lymph (Figures 3A,B). This may suggest an imbalance between CCL21-mediated retention and S1P-mediated exit signals in the enlarged LN. Measurement of total CCL21 levels by ELISA that reflects the intracellular or secreted (inactive) CCL21 and the extracellular or gradient-forming (active) CCL21 revealed a significant increase in this chemokine in LN from apoE^−/−^ mice (Figure 6B). Microscopic examination of CCL21 distribution in LN from apoE^−/−^ mice by immunostaining revealed lower levels of CCL21 associated with non-endothelial cells which might reflect its continuous secretion but higher levels of active CCL21 bound to the basement membrane of the dilated cortical and medullary sinuses but also of LYVE-1^−^ vessels (Figure 6C). Together, this finding suggest that CCL21 is more actively released in LN of apoE^−/−^ mice compared to WT mice. Moreover, quantification of S1P in lymph from WT and apoE^−/−^ mice by mass spectrometry revealed a significant decrease in lymph S1P levels in dyslipidemic mice (Figure 6D). This reduction in lymph S1P in apoE^−/−^ mice likely resulted from decreased production in LN as supported by reduced expression of LN *Sphk1* but also from increased S1P degradation by S1P lyase whose expression was markedly increased in apoE^−/−^ mice LN (Figure 6E). Together these data indicate that the decreased egress of lymphocyte from hypertrophic LN observed in apoe^−/−^ mice is due to reduced S1P exit signal and conversely, increased CCL21 retention signal.

### Reversing Dyslipidemia in apoE^−/−^ Restores Lymphocyte Egress

**Figure 7:**
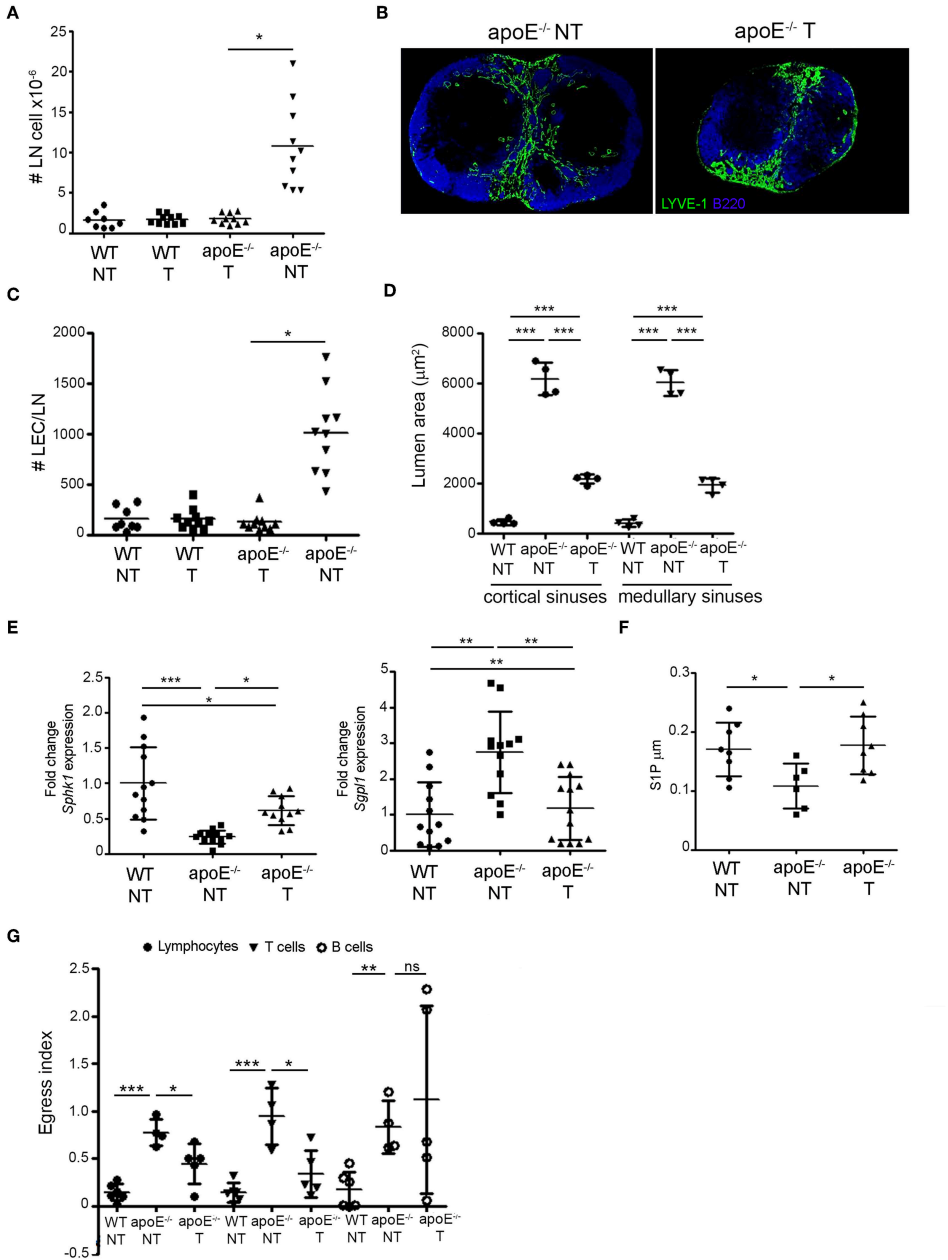
Reversing dyslipidemia improves lymphocyte egress and LN hypertrophy. **(A)** WT and apoE^−/−^ mice were treated with eztimibe (treated, T) or vehicle (non-treated, NT) and LN cellularity expressed as fold change over WT mice was determined. *n* = 8–10 mice per group; *p < 0.05. **(B)** Immunoreactivity for B220 and LYVE-1 was examined in LN sections from NT and T apoE^−/−^ mice. Images are representative of three independent experiments (*n* = 3–5). **(C)** The number of LECs was determined in LN suspensions from NT WT, NT and T apoE^−/−^ mice by flow cytometry and expressed as fold change over baseline. *n* = 8–10; *p < 0.05. **(D)** Lumen area of cortical and medullary sinuses was determined on LN sections NT WT, NT, and T apoE^−/−^ mice. *n* = 4 mice per group; ***p < 0.0005. **(E)** Quantitative RT-PCR analysis was performed on whole LN RNA to examine expression of *Sph1k* and *Spgl1* in NT WT, NT, and T apoE^−/−^ mice. Data is pooled from three independent experiments with 4 mice per group in each experiment; *p < 0.05; **p < 0.005; ***p < 0.0005. **(F)** S1P content was analyzed in efferent lymph of NT WT, NT and T apoE^−/−^ mice by mass spectrometry. Data is pooled from three independent experiments with 3–4 mice per group in each experiment **(G)** Egress of adoptively transferred CD45.1 lymphocytes, T and B cell from NT WT, NT and T apoE^−/−^ mice was examined. *n* = 4–6 mice per group; *p < 0.05; **p < 0.005; ***p < 0.0005.

Next, we tested whether reduction of circulating cholesterol levels in apoE^−/−^ mice could restore lymphocyte egress. Consistent with our and others work (34, 40, 41) daily gavage of ezetimibe, a FDA approved cholesterol-lowering drug, reduced total plasma cholesterol levels in apoE^−/−^ mice but did not affect plasma cholesterol levels in WT mice (Table 1). Following ezetimibe treatment, LN cellularity was significantly reduced in apoE^−/−^ mice compared to untreated apoE^−/−^ mice whereas LN cellularity in WT mice was not affected (Figure 7A). In ezetimibe treated apoE^−/−^ mice, lymphangiogenesis was reduced (Figure 7B) and the number of LECs was reversed to WT LEC number (Figure 7C). Since ezetimibe did not have any effect in WT mice, vehicle-treated WT mice were used as control for comparison with vehicle-treated apoE^−/−^ mice in the subsequent experiments. Ezetimibe treatment also affected the dilation of lymphatic sinuses in apoE^−/−^ mice. Indeed, the lumen area of cortical and medullary sinuses was not equivalent to WT mice but was significantly improved over untreated apoE^−/−^ mice (Figure 7D). Furthermore, the expression of *Sgpl1* in LN from ezetimibe treated apoE^−/−^ mice was improved to WT expression whereas the expression of *Sphk1* was significantly increased compared to untreated apoE^−/−^ mice (Figure 7E). Importantly, these changes in ezetimibe treated apoE^−/−^ mice resulted in the restoration of lymph S1P levels comparable to WT controls (Figure 7F). In contrast, the expression of CCL21 surrounding the cortical and medullary in LN from ezetimibe treated apoE^−/−^ mice was not significantly different compared to untreated apoE^−/−^ mice (data not shown). Nevertheless, the amelioration of cortical and medullary sinuses dilatation and the reversal of LEC numbers and lymph S1P levels in ezetimibe treated apoE^−/−^ mice were sufficient to override CCL21 retention signal since lymphocyte egress from LN was partially restored in these mice, increasing lymphocyte egress index by 50% (Figure 7G). This effect was mainly due to the improvement of T cell egress but not B cell.

**Table 1:** Effect of ezetimibe on total cholesterol.

**Mice**	**Treatment**	**Total cholesterol**
Wild-type	Vehicle	34.95 ± 4.72
	Ezetimibe	22.6 ± 2.49
Apoe^−/−^	Vehicle	1882 ± 188.70
	Ezetimibe	791.7 ± 111.50^***^

*Values are mean ± SEM*.

*** *p < 0.0001*.

## Discussion

Here, we elucidate the mechanisms whereby hypercholesterolemia in mouse models leads to the accumulation of lymphocytes in skin draining LNs and subsequent LN hypertrophy. We show that the increased number of lymphocytes does not result from increased proliferation or input of lymphocyte within the enlarged LN but from the severe impairment of lymphocytes to emigrate from those LN via efferent lymph. The adoptive transfer experiments used to determine lymphocyte egress also revealed that defects in extrinsic microenvironment surrounding lymphocytes rather than intrinsic defects in lymphocytes account for the impaired lymphocyte egress in apoE^−/−^ mice compared to WT mice. Lymphatic vessels are essential for lymphocyte trafficking. We showed previously that skin lymphatic transport is severely compromised in hypercholesterolemic apoE^−/−^ and Ldlr^−/−^ mice which also exhibit LN hypertrophy and impaired skin DC migration (34, 42). Therefore, we hypothesized that hypercholesterolemia may alter the lymphatic sinuses of the activated LN.

Consistent with this hypothesis, we found a pronounced expansion of the lymphatic network in LNs from apoE^−/−^ mice as denoted by a >20-fold increase in LEC numbers and the expression of proliferative marker Ki67. This expansion affects predominantly the cortical and medullary sinuses. Further microscopic examination revealed structural abnormalities in this expanded sinuses network including tortuous morphology, severe dilation and local loss of LYVE-1 expression. Such “mosaic” vessels have also been observed in various tumors as a result of extensive intra-tumoral lymphangiogenesis (39, 43) and have been shown to be “leaky” and as a consequence poor lymph carrier (43). Thus, it is possible that these structural abnormalities in the overly expanded sinuses of apoE^−/−^ LN may lead to “leaky” sinuses and disturb efferent lymph flow which is critical for optimal lymphocyte transport from the LN (44). Altered efferent flow may also diminish the transport of lymphocytes within efferent lymph which is consistent with the poor number of lymphocyte collected in efferent lymph from apoE^−/−^ mice and conversely, increase the opportunity for CCL21 to attract lymphocytes back to the LN parenchyma. Notably, numerous adoptively transferred lymphocytes in apoE^−/−^ mice were found within and surrounding cortical and medullary sinuses where CCL21 was more abundant compared to WT mice. Although the measure of lymph flow will require further investigations which are beyond the scope of this study, our previous data on skin lymphatics in apoE^−/−^ mice showing that they are leaky and exhibit abnormal valve (34) support the idea of altered efferent lymph flow in these hypercholesterolemic mice. The extensive remodeling of lymphatic sinuses observed in apoE^−/−^ mice was also associated with a significant decrease in lymph S1P levels. This decrease may result from a reduced local production of S1P by LECs as supported by lower expression of S1PK but also from an increased degradation by S1P lyase whose expression was significantly higher in apoE^−/−^ LN compared to WT LN. However, we cannot exclude the possible contribution of systemic abnormalities in sphingolipids as shown in a lipidomic study by Chen et al., reporting elevated plasma levels of sphingolipids and abnormal sphingolipid metabolism in apoE^−/−^ mice (45).

Finally, we provide evidence that hypercholesterolemia accounts in part for the impaired lymphocyte egress, extensive remodeling of lymphatic sinuses and decrease in lymph S1P levels as these defects were restored by reversing hypercholesterolemia with the lowering-cholesterol drug ezetimibe. This is in line with our previous report demonstrating that ezetimibe improves lymphatic drainage and skin dendritic cell migration in both apoE^−/−^ and Ldlr^−/−^ mouse models (34). By analogy to arterial alterations induced by hypercholesterolemia, the accumulation of lipoprotein including LDL and oxidized LDL may compromise the structural integrity of lymphatic vessels by for example altering the expression of tight junction assembly (46) or may induce lymphatic dysfunction by affecting smooth muscle contraction of efferent lymphatic vessels through the modulation of nitric oxide levels (47). However, hypercholesterolemia in apoE^−/−^ mouse is often associated with systemic inflammation including in skin (26, 27, 33). Therefore, inflammation that can affect lymphatic vessel structure and function may also account for lymphatic alterations and impaired lymphocyte trafficking. Although ezetimibe has been shown to reduce atherosclerosis progression mainly through its effect on plasma LDL cholesterol by inhibiting its absorption by the intestine (40, 41), it is possible that ezetimibe may indirectly affect inflammation associated with hypercholesterolemia in apoE^−/−^ mice by reducing LDL cholesterol and subsequently inflammatory modified LDL. Therefore, it would be interesting to investigate whether anti-inflammatory strategies alone without affecting hypercholesterolemia would be sufficient to improve lymphocyte egress and lymphatic function in apoE^−/−^ mice. That the improvement of lymphocyte egress in apoE^−/−^ mice by ezetimibe treatment resulted from the improved T cell egress but not that of B cell suggests that the egress of B cells may rely on additional signals than S1P. In fact, most of the knowledge on lymphocyte egress is based on studies on T cell egress.

It is now apparent that the expansion of lymphatic vessels in LN can modulate the immune response during inflammation (38, 48, 49). All of these studies however focused on lymphangiogenesis occurring at early phases of inflammation. More recently, we reported a biphasic remodeling of lymphatic vessels during the course of inflammation, the subcapsular sinuses being expanded first followed by the cortical and medullary sinuses (23). Notably, this differential remodeling is biologically and functionally important. Indeed, the early expansion of subcapsular sinuses enhances DC migration from the periphery into the inflamed LN (38) whereas the expansion of cortical and medullary sinuses at later phases of inflammation reestablishes the steady-state egress of lymphocytes from those LNs (23). Therefore, the increased accumulation of lymphocytes into LNs during inflammation has to be accompanied by a proportional increase in lymphocyte exit in the efferent lymph to prevent LN hypertrophy. Here, we provide evidence that under certain circumstances such as chronic inflammation associated with hypercholesterolemia, the aberrant expansion of cortical and medullary sinuses can conversely inhibit the emigration of lymphocyte from the inflamed LNs leading to subsequent LN hypertrophy.

Activated antigen-specific lymphocytes must leave the LN to migrate into the effector sites in order to exert their appropriate immune responses (9). Naïve lymphocyte must also exit the LN via efferent lymph to reach the blood circulation for immune surveillance. Therefore, the impairment of lymphocyte egress is expected to affect the normal function of both naïve and effector lymphocytes and subsequently, compromised immune responses. This scenario is likely relevant to hypercholesterolemic mouse models. Indeed, apoE^−/−^ and Ldlr^−/−^ mice show impaired priming when immunologically challenged (33) and reduced capacity for clearance of bacteria (29, 30, 50), fungi (28), and virus (31). In addition, this increased susceptibility to infection does not occur in young apoE^−/−^ mice, but increases with age (31, 51), consistent with the later onset of LN hypertrophy and decreased lymphocyte egress. Thus, the increased susceptibility to infection in hypercholesterolemic mice may not only result from impaired DC migration from peripheral tissue into the draining LN as we proposed previously (33) but also from the impairment in efferent lymphatic emigration of lymphocytes from the enlarged LN. This is supported by the study of Ludewig et al. (31) showing that apoE^−/−^ mice infected with LCMV exhibit impaired migration of virus specific CD8 cytotoxic T cells into the blood and liver.

In conclusion, we provide evidence that dyslipidemia severely compromises the efferent lymphatic emigration of lymphocyte from LN which subsequently leads to LN hypertrophy by altering the structure and function of lymphatic vessels. Since LN hypertrophy is often associated with chronic inflammatory and autoimmune diseases such as systemic lupus erythematosus and rheumatoid arthritis, our findings may also be relevant to other chronic diseases than those related to dyslipidemia. This study further illustrates the importance of maintaining healthy lymphatic vessel for optimal immunity.

## Data Availability

All datasets generated for this study are included in the manuscript and the Supplementary Files.

## Author Contributions

SL, MT, YL, KT, FT, PN, MW, CT, and VA designed the study and developed the methodology. SL, MT, YL, KT, FT, PN, and CT performed and analyzed the experiments and VA wrote the manuscript.

### Conflict of Interest Statement

The authors declare that the research was conducted in the absence of any commercial or financial relationships that could be construed as a potential conflict of interest.
