# Supplementary figures and images for: Halted Lymphocyte Egress via Efferent Lymph Contributes to Lymph Node Hypertrophy During Hypercholesterolemia
Source: Front Immunol. 2019 Mar 27;10:575. doi: 10.3389/fimmu.2019.00575 (PMC6446103; doi:10.3389/fimmu.2019.00575)

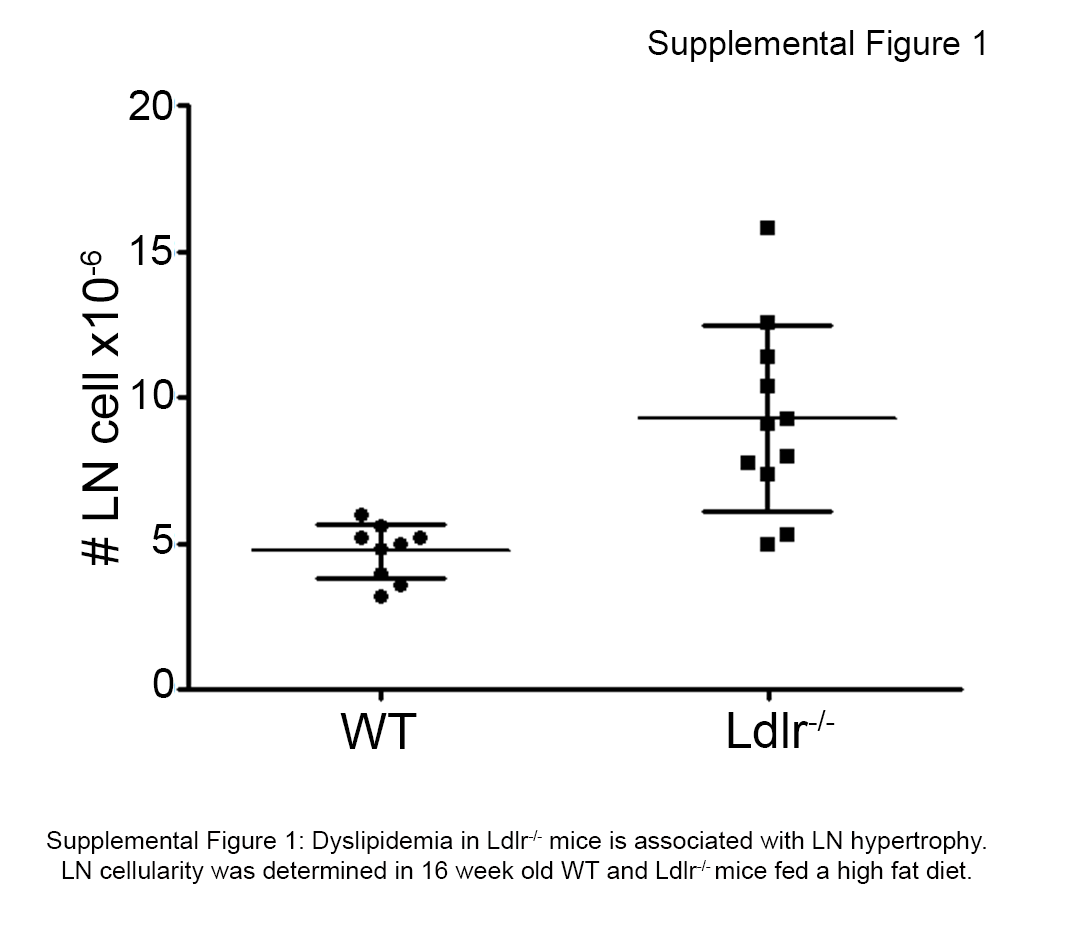

Supplement: Supplementary file 1 [file Image_1.tif]
